# Radiotherapy after Radical Prostatectomy: Treatment Recommendations Differ between Urologists and Radiation Oncologists

**DOI:** 10.1371/journal.pone.0079773

**Published:** 2013-11-04

**Authors:** Luke T. Lavallée, Dean Fergusson, Ranjeeta Mallick, Renée Grenon, Scott C. Morgan, Franco Momoli, Kelsey Witiuk, Chris Morash, Ilias Cagiannos, Rodney H. Breau

**Affiliations:** 1 Division of Urology, Department of Surgery, The Ottawa Hospital, University of Ottawa, Ottawa, Ontario, Canada; 2 Clinical Epidemiology Program, Ottawa Hospital Research Institute, Ottawa, Ontario, Canada; 3 Children’s Hospital of Eastern Ontario Research Institute, Ottawa, Ontario, Canada; 4 Division of Radiation Oncology, The Ottawa Hospital, University of Ottawa, Ottawa, Ontario, Canada; National University of Ireland Galway, Ireland

## Abstract

**Purpose:**

There is no consensus on optimal use of radiotherapy following radical prostatectomy. The purpose of this study was to describe opinions of urologists and radiation oncologists regarding adjuvant and salvage radiotherapy following radical prostatectomy.

**Methods:**

Urologists and genitourinary radiation oncologists were solicited to participate in an online survey. Respondent characteristics included demographics, training, practice setting, patient volume/experience, and access to radiotherapy. Participant practice patterns and attitudes towards use of adjuvant and salvage radiotherapy in standardized clinical scenarios were assessed.

**Results:**

One hundred and forty-six staff physicians participated in the survey (104 urologists and 42 genitourinary radiation oncologists). Overall, high Gleason score (Gleason 7 vs. 6, RR 1.37 95% CI 1.19-1.56, p<0.0001 and Gleason 8-10 vs. 6, RR 1.56 95% CI 1.37-1.78, p<0.0001), positive surgical margin (RR 1.43 95% CI 1.26-1.62, p<0.0001), and extraprostatic tumour extension (RR 1.16 95% CI 1.05-1.28, p<0.002) conferred an increased probability of recommending adjuvant radiotherapy. Radiation oncologists were more likely to recommend adjuvant radiotherapy across all clinical scenarios (RR 1.48, 95% CI 1.39, 1.60, p <0.001). Major differences were found for patients with Gleason 6 and isolated positive surgical margin (radiotherapy selected by 21% of urologists vs. 70% of radiation oncologists), and patients with extraprostatic extension and negative surgical margins (radiotherapy selected by 18% of urologist vs. 57% of radiation oncologists).

**Conclusions:**

Urologists and radiation oncologists frequently disagree about recommendation for post-prostatectomy adjuvant radiotherapy. Since clinical equipoise exists between adjuvant versus early salvage post-operative radiotherapy, support of clinical trials comparing these approaches is strongly encouraged.

## Introduction

Approximately 40% of patients diagnosed with prostate cancer will be treated with radical prostatectomy and approximately 15-35% will develop a detectible serum prostate-specific antigen (PSA) following surgery [1-7]. A positive surgical margin, extraprostatic tumour extension, and seminal vesicle tumour invasion occur in approximately 34%, 24%, and 9% of patients and have been associated with increased risk of biochemical recurrence following prostatectomy [7-13]. The optimal management of patients who have adverse pathologic features after prostatectomy is unknown but may include observation, adjuvant radiotherapy, and androgen deprivation therapy [14]. 

Traditionally, clinicians may have been more likely to treat patients with androgen deprivation if clinicopathologic features were associated with high risk of systemic disease (e.g. immediately detectable PSA, seminal vesicle invasion, or high Gleason score) and more likely to treat patients with adjuvant or salvage radiotherapy if features were associated with high risk of isolated localized disease (e.g. late PSA recurrence, positive surgical margin, extraprostatic tumour extension, and low Gleason score) [15]. However, recent randomized trials in North America and Europe call into question these clinical assumptions about management of post-prostatectomy patients. These trials (SWOG 8794, EORTC 22911, and ARO 06-02) revealed that adjuvant pelvic radiotherapy benefits patients who are at high risk of local and systemic recurrence (e.g. immediately detectable post-operative PSA, seminal vesicle invasion, high Gleason score) [16-18]. 

While indications for pelvic radiotherapy may have changed, questions remain about the timing of treatment. Adjuvant radiotherapy is offered to patients prior to PSA recurrence while salvage radiotherapy reserves treatment, and its associated side effects, for men with proven biochemical recurrence. Randomized trials of adjuvant radiotherapy versus observation reveal a reduction in biochemical recurrence (SWOG 8794, EORTC 22911, ARO 06-02) and prolonged survival (SWOG 8794) while observational studies suggest salvage radiotherapy versus observation reduces progression and prolongs survival [19,20]. The purpose of this study was to determine which clinical and pathological factors were associated with the recommendation of adjuvant and salvage radiotherapy by urologists and radiation oncologists.

## Methods

### Recruitment

In October 2011, Canadian Urological Association (CUA) members and genitourinary radiation oncologists from the Canadian Association of Radiation Oncology (CARO) were invited to participate in an online survey. CUA members were contacted through the CUA listserv and radiation oncologist emails were available through the CARO member directory. A second request was made one month following the original invitation. E-mail recipients were provided with a hyperlink to an online survey that populated a secure database. The survey was closed on November 7^th^, 2011. Institutional ethics approval was obtained for this survey from the Ottawa Hospital Research Ethics Board prior to study commencement.

### Survey

Participants were asked to provide demographic information including age, geographic location of practice, specialty (urology or radiation oncology), practice type (academic or community), years in practice, sub-specialty training, number of prostate cancer patients assessed per year, and access to radiotherapy.

Respondents were asked to rate how various clinical and pathologic variables independently influenced their recommendation for adjuvant and salvage radiotherapy via Likert items. Clinical variables included patient age, urinary continence, and erectile function. Pathologic variables included extraprostatic tumour extension, seminal vesicle tumour invasion, lymph node metastases, pre- and post-operative PSA concentrations, Gleason score, pathologic stage, and surgical margin status. 

Respondents were provided with a standardized case scenario of a healthy 60 year old male 3-months post radical prostatectomy with an undetectable PSA. They were asked to recommend for or against adjuvant radiotherapy for this patient given varied Gleason score, pathologic stage, and surgical margin status.

### Statistical Methods

Survey responses were summarized with frequencies and percentages. Associations between clinician/patient characteristics and choice of adjuvant radiotherapy were calculated using logistic regression. Log binomial regression was used to calculate relative risks (RR) using the proc genmod procedure in SAS. RR are presented with 95% confidence intervals (CI). Candidate predictor variables included clinician characteristics (age, specialty, fellowship training, practice location, and access to radiotherapy) and pathologic factors (Gleason grade, surgical margin status, and pathologic stage). Each predictor variable was assessed as a categorical variable. The primary outcome was the respondent’s decision to recommend *adjuvant radiotherapy* after radical prostatectomy in the provided clinical scenario. A-priori independent variables for the clinical scenario model were Gleason grade, tumour stage, surgical margin status, and medical specialty. P-values < 0.05 were considered statistically significant.

## Results

From 586 listed emails, 146 (25%) staff physicians participated in the survey and were included in the analysis. 104 (22%) of solicited urologists and 42 (40%) of solicited genitourinary radiation oncologists completed the survey. The majority of radiation oncologists (n=39, 93%) and approximately half of urologists (n=86, 59%) indicated they practiced in an academic institution. Sub-specialty training in genitourinary oncology was reported in 51 (35%) respondents. The mean number of years in practice was 14 years (SD 10.3). Full demographic characteristics are presented in Table 1. 

**Table 1:** Characteristics of urologist and radiation oncologists who participated in the Pelvic Radiotherapy after Radical Prostatectomy Survey.

	**Overall Frequency (%)**	**Urologist Frequency (%)**	**Radiation Oncologist Frequency (%)**
**Specialty**	146 (100)	104 (70.8)	42 (28.6)
**Age**			
30-39	46 (31.3)	33 (32.3)	13 (30.1)
40-49	44 (29.9)	26 (25.5)	18 (42.9)
50-59	32 (21.8)	26 (25.5)	6 (14.3)
≥ 60	23 (15.7)	17 (16.6)	5 (11.9)
**Type of practice**			
Academic	86 (58.5)	46 (45.1)	39 (92.8)
Community	59 (40.1)	56 (54.9)	3 (7.1)
**Sub-specialty (fellowship) in genitourinary oncology**			
No	93 (63.3)	74 (73.3)	19 (45.2)
Yes	51 (34.7)	27 (26.7)	23 (54.8)
**# of prostate cancer patients treated annually**			
>50	103 (70.1)	68 (65.3)	34 (81.0)
31-50	22 (15.0)	19 (18.3)	3 (7.1)
10 - 30	18 (12.2)	15 (14.4)	3 (7.1)
<10	4 (2.7)	2 (1.9)	2 (4.8)
**Radiotherapy access rating**			
Excellent	119 (81.6)	84 (80.8)	35 (83.3)
Poor / Average	27 (18.4)	20 (19.2)	7 (16.7)

(Note: all respondents did not answer all questions accounting for differences in the frequency between categories.)

A majority of urologists (n=49, 58%) and radiation oncologists (n=36, 90%) reported that recent randomized trials have changed the way they manage patients after radical prostatectomy. When asked their opinion regarding the value of adding androgen deprivation therapy (ADT) to adjuvant or salvage radiotherapy, 29 (35%) urologists and 13 (32%) radiation oncologists reported believing it is likely beneficial and 44 (52%) urologists and 22 (55%) radiation oncologist would recommend it to high risk patients. A large proportion of urologists (n=37, 44%) and radiation oncologists (n=33, 82%) believed more research is required to evaluate the benefit and harm of ADT when used in conjunction with radiotherapy following prostatectomy. 

**Figure 1:**
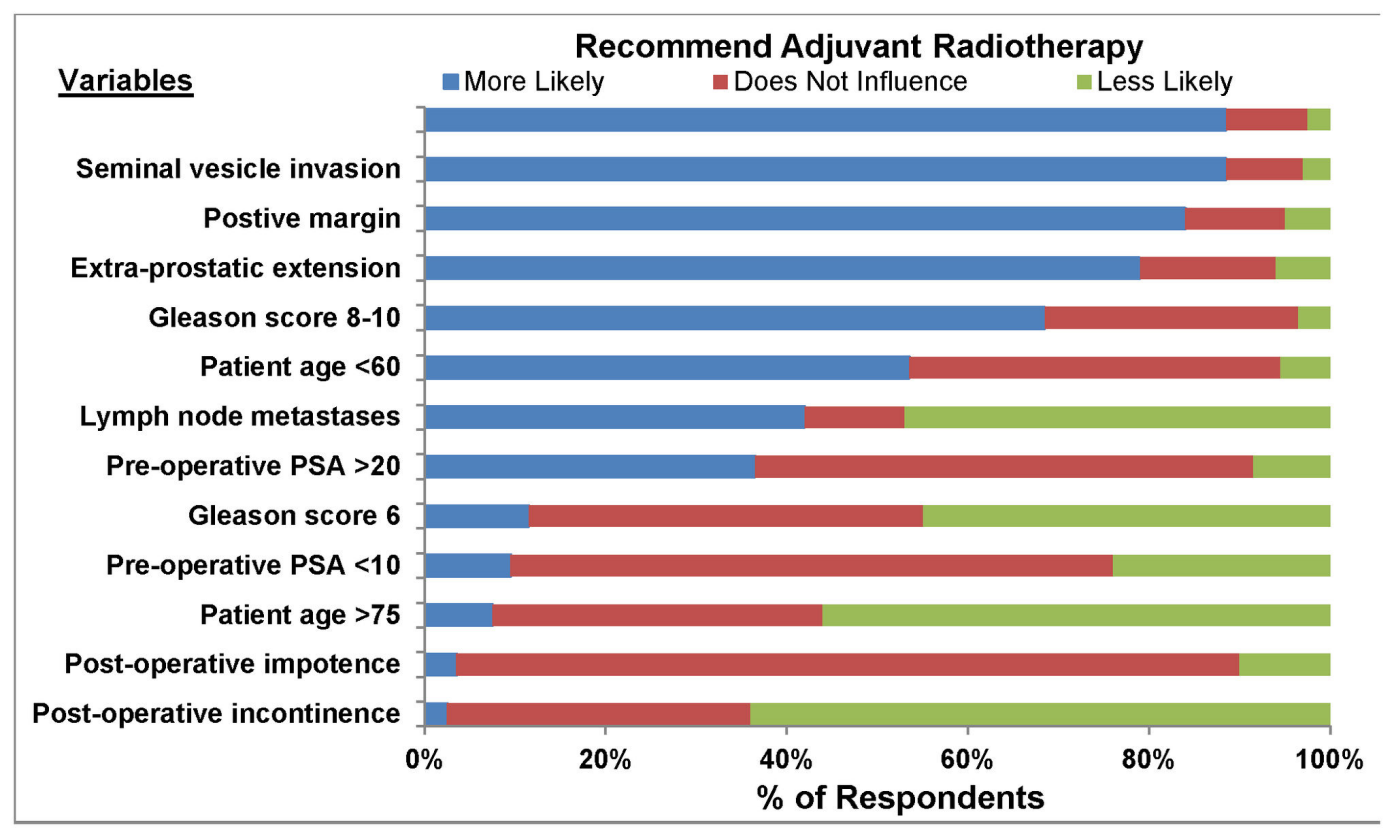
The influence of clinical and pathologic variables on the likelihood of recommending *adjuvant radiotherapy* (PSA units are ng/mL).

**Figure 2:**
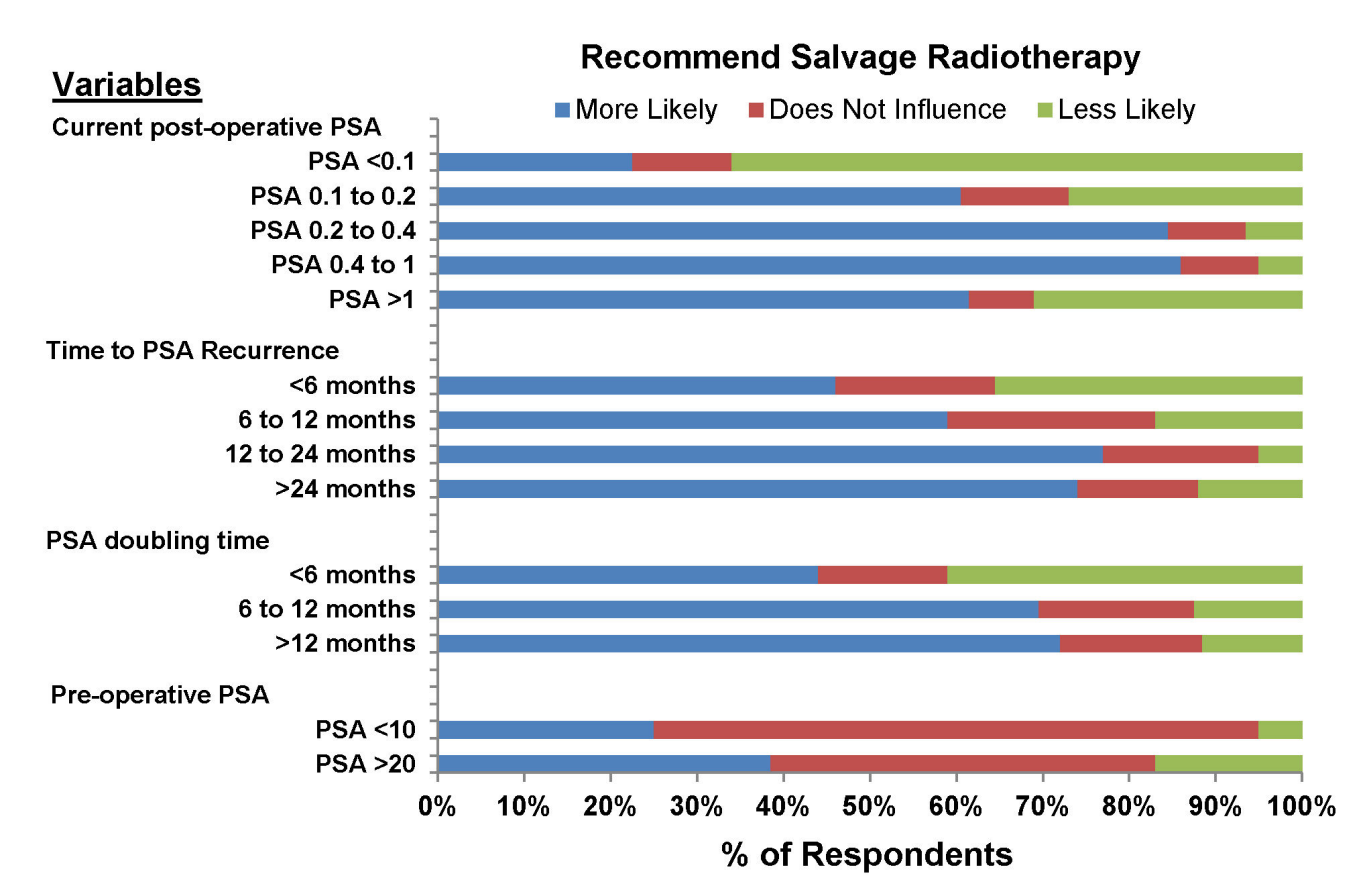
The influence of pre-operative and post-operative PSA characteristics on the likelihood of recommending *salvage* *radiotherapy*. Pre-operative PSA refers to the most recent PSA prior to radical prostatectomy (PSA units are ng/mL).

The influence of clinical and pathologic factors on the recommendation for adjuvant or salvage radiotherapy is presented in Figures 1 and 2. Positive surgical margin, mutlifocal/large positive surgical margin, extraprostatic extension, seminal vesicle invasion, Gleason score 8-10, and patient age <60 years were associated with the recommendation for adjuvant radiotherapy. Gleason score 6, pre-operative PSA <10, age >75 years, post-operative incontinence, and post-operative impotence influenced most respondents to recommend against adjuvant radiotherapy. The same factors were associated with a preference for or against salvage radiotherapy (data not shown). In addition, other clinical parameters supported salvage radiotherapy, including higher post-operative PSA values up to a threshold of 1, longer time to PSA recurrence, and longer PSA doubling time (Figure 2). 

**Figure 3:**
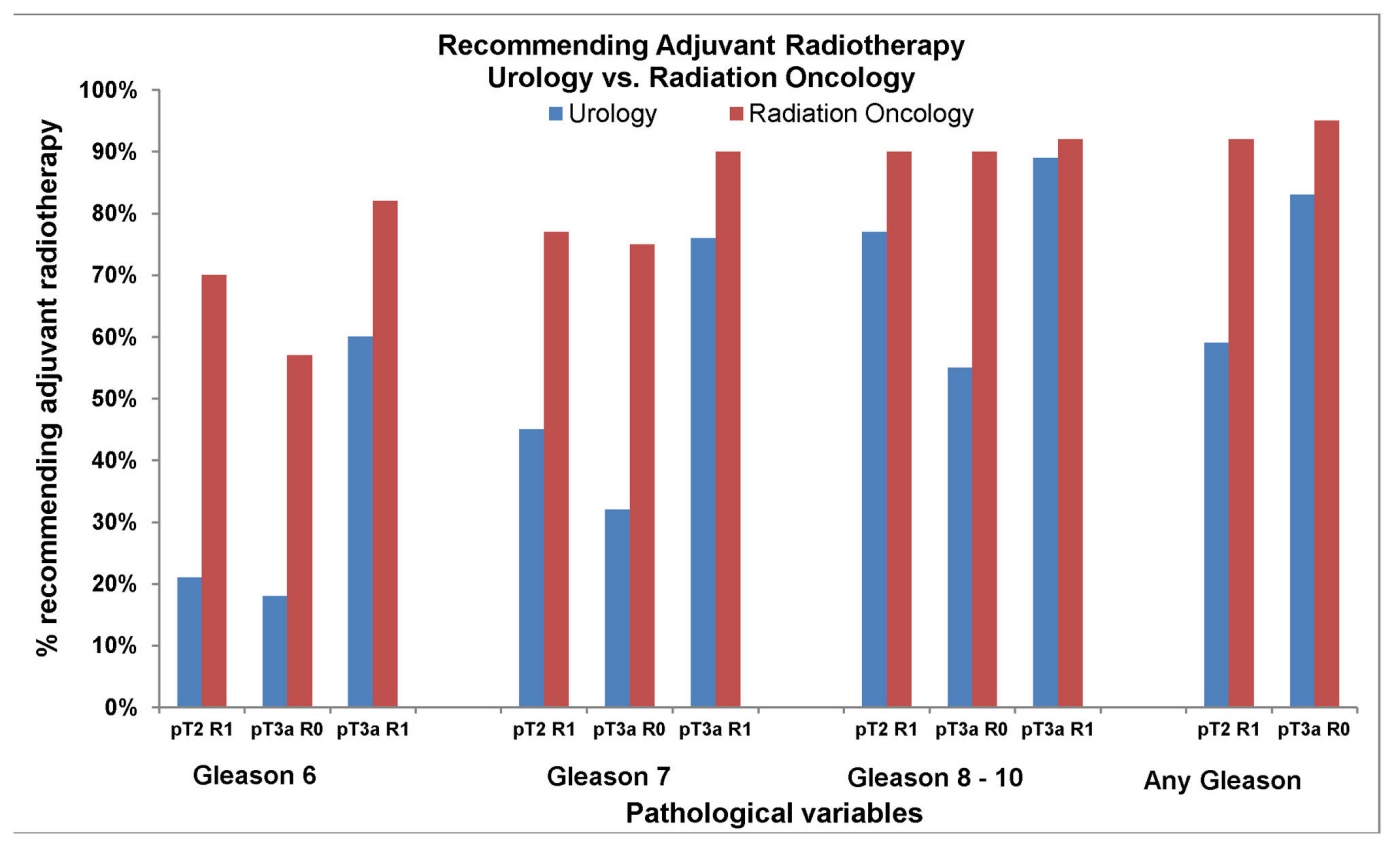
Respondents were asked to indicate if they recommend adjuvant radiotherapy for a fit 60 year old following a radical prostatectomy with an undetectable post-operative PSA given specific pathological findings. This Figure describes the responses of urologists and radiation oncologists for each set of pathological variables.

Large differences were identified in the opinions of radiation oncologists and urologists regarding recommendations for adjuvant radiotherapy when pathologic variables were placed in the context of a standardized clinical scenario (Figure 3). Seventy percent of radiation oncologists recommend adjuvant radiotherapy in a patient with Gleason 6 stage pT2 and a positive surgical margin (Gleason 6 pT2R1) compared to 21% of urologists. For all presented scenarios, radiation oncologists were more likely to recommended adjuvant radiotherapy compared to urologists (Figure 3). 

Overall, radiation oncologists were 48% more likely to recommend adjuvant radiotherapy compared to urologists (Absolute difference 27%, unadjusted RR 1.48, 95% CI 1.39, 1.60, p <0.001). Of the remaining clinical factors evaluated, only physician age had a significant impact on the recommendation for adjuvant radiotherapy with older physicians being less likely to recommend radiotherapy (RR 0.96 95% CI 0.93, 0.99, p =0.03). Practice setting, access to radiotherapy, and genitourinary oncology fellowship training did not strongly influence the probability of recommending radiotherapy (Table 2).

**Table 2:** Unadjusted associations of clinician characteristics and pathologic variables and the recommendation for adjuvant radiotherapy.

	Overall*		**Urology**		**Radiation Oncology**	
	**Relative Risk (95% CI)**	**p-value**	**Relative Risk (95% CI)**	**p-value**	**Relative Risk (95% CI)**	**p-value**
**Age** (n = 127)						
30-39 (ref.)	1.0	-	1.0	-	1.0	-
40-49	1.17 (1.06-1.28)	0.001	1.14 (0.99-1.31)	0.09	1.07 (0.96-1.18)	0.20
50-59	0.98 (0.88-1.10)	0.78	0.97 (0.83-1.13)	0.70	1.19 (1.08-1.31)	0.0007
≥60	0.86 (0.74-0.99)	0.05	0.92 (0.76-1.12)	0.42	0.72 (0.55-0.95)	0.02
**Fellowship Training**						
(n = 127)						
Yes vs. No	0.94 (0.86-1.02)	0.11	0.77 (0.67-0.89)	0.0003	0.92 (0.84-1.0)	0.05
**Gleason score**						
(n = 128)						
7 vs.6	1.38 (1.20-1.59)	<0.0001	1.60 (1.29-1.98)	<0.0001	1.16 (1.0-1.34)	0.05
8-10 vs. 6	1.81 (1.60-2.05)	<0.0001	2.33 (1.92-2.82)	<0.0001	1.30 (1.14-1.49)	<0.0001
8-10 vs. 7	1.31 (1.19-1.44)	<0.0001	1.45 (1.26-1.67)	<0.0001	1.13 (1.0-1.25)	0.03
**Surgical Margin**						
(n =129)						
R1 vs. R0	1.35 (1.23-1.48)	<0.0001	1.60 (1.39-1.85)	<0.0001	1.08 (0.99-1.19)	0.1
**Stage** (n = 129)						
3a vs. 2	1.09 (0.98-1.26)	0.10	1.14 (0.98-1.32)	0.09	1.03 (0.92-1.15)	0.63
3b vs. 2	1.35 (1.21-1.50)	<0.0001	1.48 (1.27-1.74)	<0.0001	1.19 (1.06-1.32)	0.002
3b vs. 3a	1.24 (1.14-1.35)	<0.0001	1.31 (1.16-1.47)	<0.0001	1.16 (1.06-1.26)	0.0007
**Practice Setting**						
(n = 127)						
Community vs. Academic	0.84 (0.77-0.91)	<0.0001	1.07 (0.95-1.20)	0.29	0.95 (0.79-1.14)	0.56
**Access to Radiotherapy**						
(n = 129)						
Excellent vs. Poor/average	1.04 (0.93-1.15)	0.50	1.16 (0.98-1.37)	0.08	0.89 (0.82-0.97)	0.01

* **10 respondents did not provide specialty information**

Pathologic variables (Gleason score, surgical margin, and pathologic stage) were associated with the recommendation for adjuvant radiotherapy (Table 2). A Gleason score of 8-10 on a radical prostatectomy specimen conferred a relative risk of 1.81 when compared to a Gleason score of 6 (95% CI 1.60, 2.05, p < 0.0001). 

To adjust for potential confounders, multivariable analysis was performed to determine the independent associations between each predictor variable and the recommendation for adjuvant radiotherapy (Table 3). Pathologic findings (Gleason score, surgical margin status, pathologic stage) and specialty (radiation oncologist) conferred an increased probability of recommending radiotherapy.

**Table 3:** Adjusted multivariate analysis of the influence of specialty (radiation oncology vs. urology) and pathologic variables on the relative risk of recommending adjuvant radiotherapy.

	**Relative Risk**	**95% Confidence Interval**	**P-value**
**Gleason 7 vs. 6**	1.37	1.19, 1.56	<0.0001
**Gleason 8 to10 vs. 6**	1.56	1.37, 1.78	<0.0001
**Gleason 8 to10 vs. 7**	1.14	1.04, 1.25	0.004
**Surgical Margin R1 vs. R0**	1.43	1.26, 1.62	<0.0001
**Stage 3a vs. 2**	1.16	1.05, 1.28	0.002
**Specialty:**			
**Radiation oncology vs. Urology**	1.26	1.15, 1.38	<0.0001

The model adjusts for Gleason grade, surgical margin status, stage, and specialty*. (n = 128 for the multivariate model)

* *Seminal vesicle invasion was not included in the analysis because it was not examined independently in the survey.*

## Discussion

This survey explored the practice patterns and opinions of two different specialty groups involved in the management of patients with prostate cancer, urologists and radiation oncologists, and identified factors that influence recommendations for post-operative radiotherapy. For every pathological variable examined, radiation oncologists were more likely to recommend adjuvant radiotherapy than were urologists with a mean absolute difference of 27% and relative difference of 48%. There were clinical scenarios where urologists and radiation oncologists agreed; specifically, both commonly recommended adjuvant radiotherapy in patients with extraprostatic tumour extension and a positive surgical margin, high Gleason score and a positive surgical margin, or seminal vesicle invasion. Conversely, there was lack of consensus for patients with low Gleason score and an isolated positive surgical margin, and for patients with extraprostatic extension and negative surgical margin. The influence of medical specialty on treatment recommendations was striking and is a trend that has been previously observed amongst urologists and radiation oncologists when selecting primary treatment of prostate cancer [21].

Standardized scenarios in the survey represented patients that would have met inclusion criteria for entry into SWOG 8794 [16]. The SWOG 8794 trial randomized post-prostatectomy patients to adjuvant radiotherapy or observation and revealed improved cancer-specific and overall survival in the post-operative radiotherapy arm. Subset analyses revealed a relative benefit of post-operative adjuvant radiotherapy in patients with positive margins, T3 disease, and high Gleason scores [16]. The findings from SWOG 8794 were not consistently observed in two other randomized trials [14]. EORTC 22911 and ARO 96-02 found that adjuvant radiotherapy caused a reduction in the risk of PSA recurrence primarily in patients with positive surgical margins [17,22]. Reporting of EORTC 22911 after more than 10 years of follow up has not demonstrated improvement in clinical progression or overall survival (81% vs. 77%, p > 0.1) and at 54 months follow-up, ARO 96-02 did not have a sufficient number of events to make definitive conclusions regarding systemic progression (metastases n = 9) or overall survival (death n = 13) [17,23]. It would appear that radiation oncologists are influenced by the overall relative benefit observed in SWOG 8794 and therefore recommend adjuvant radiotherapy to patients who meet the inclusion criteria of that study. Alternatively, urologists were less likely to recommend adjuvant radiotherapy, perhaps due to concerns of overtreatment and familiarity with the often indolent natural history of this disease following radical prostatectomy [24]. 

Examining each variable independently, a large proportion of physicians surveyed were more likely to recommend adjuvant post-operative radiotherapy in patients with extraprostatic disease, high Gleason score, seminal vesicle invasion, and positive surgical margins. It is interesting to note that surgical margin status appeared to influence urologists much more than radiation oncologists (RR 1.6 vs. 1.08). Taken as a whole, these results indicate a departure from the dogmatic approach of avoiding radiotherapy in patients with high risk of metastases (i.e. high Gleason score, immediately detectably post-operative PSA, and seminal vesicle invasion) [15]. Factors that decreased the probability of recommending radiotherapy in this survey included older patients and urinary incontinence. While we did not ask reasons for these choices, physicians probably are less likely to recommend radiotherapy in older men because they are more likely to die from a non-prostate cancer cause, and less likely to recommend radiotherapy in patients with urinary incontinence to maximize the likelihood of continence recovery [16,25]. 

Among post-operative patients who develop a detectable serum PSA, the survey identified several factors that influence the likelihood of physicians recommending salvage radiotherapy. In addition to the pathological and clinical characteristics identified for adjuvant radiotherapy, post-operative PSA kinetics strongly influenced physician opinion. Physicians were more likely to recommend radiotherapy when there was a prolonged duration between prostatectomy and detection of post-operative PSA and when the PSA doubling time was long. This finding is somewhat surprising and illustrates a misconception amongst physicians surveyed regarding the value of salvage radiotherapy for long and short PSA doubling times (PSADT). Although observational studies have reported a better prognosis for men with PSADT > 10 months, the absolute benefit of salvage radiotherapy is in fact greater in men with rapid PSADT [8,20]. Therefore a short PSADT should not deter physicians from recommending salvage radiotherapy. Physicians were less likely to recommend radiotherapy when the post-operative PSA was above 1. This is supported by observational studies reporting salvage radiotherapy to be less effective at higher PSA levels [19].

A limitation of the current literature is a lack of randomized comparison of adjuvant and salvage radiotherapy. Adjuvant radiotherapy (prior to PSA recurrence) may be better than salvage radiotherapy (when PSA becomes detectible) for several reasons. Firstly, recurrences are predominantly local in the absence of seminal vesicle invasion or lymph node metastases, therefore early local treatment may prevent metastases [25]. Furthermore, because it is given for undetectable disease, adjuvant therapy may be effective at lower radiation doses than traditional salvage [26]. Indeed, although adjuvant radiotherapy is associated with increased complications compared to observation (23.8% vs. 11.9%), health related quality of life at 5 years was observed to be better for men in SWOG randomized to surgery plus radiotherapy compared to surgery alone [27,28]. Despite favourable results in randomized trials of adjuvant radiotherapy, salvage therapy remains an attractive option because it avoids overtreatment in patients who are not destined to recur. Despite the lack of randomized data comparing early salvage radiotherapy to adjuvant radiotherapy, observational studies suggest that early salvage radiotherapy reduces PSA recurrence, distant metastases, and prostate cancer specific death [19,20]. Current use of ultra-sensitive PSA, which allows early detection of recurrence, may lessen some of the adverse effects previously reported with salvage radiotherapy when it was given for recurrent palpable disease. 

Identification of patients likely to benefit from post-operative radiotherapy is important since this intervention is associated with harm [28]. Complications of post-prostatectomy radiotherapy include urinary frequency, proctitis, rectal bleeding, urethral strictures, secondary malignancy, reduced erectile function recovery, and possibly worse continence. The fact that urologists and radiation oncologists opinions differ so significantly regarding which patients require radiotherapy, and the timing of such therapy, is striking and emphasizes the importance of clinical trials to determine optimal patient management. Two randomized trials are currently underway to compare adjuvant to early salvage radiotherapy (RADICALS – Radiotherapy and Combined Androgen Deprivation after Local Surgery, and RAVES – Radiotherapy Adjuvant vs. Early Salvage following Radical Prostatectomy) [29,30]. Until these trials are completed, the uncertainty between adjuvant and early salvage radiotherapy will continue [29,30].

This survey has limitations. We are unable to determine the true response rate since email lists were used and the activity of the email accounts could not be determined. While a wide distribution of clinician age, practice setting, and sub-specialty training was observed in the respondents, information about non-respondents was not available to assess potential biases. This survey included primarily Canadian physicians, and practice patterns may be different in other health care settings. Finally, this survey draws data from responses to realistic but fictional patient scenarios and thus cannot control for variations in respondent interpretation.

## Conclusions

The results of this survey reveal some consensus and some salient differences in the opinions of physicians regarding post-radical prostatectomy radiotherapy. Most notably, there was a large difference in the proportion of urologists and radiation oncologists who recommended radiotherapy for patients with low Gleason scores and isolated positive margins or extraprostatic extension. Since clinical equipoise exists between adjuvant and early salvage post-operative radiotherapy, support of clinical trials comparing these two approaches is strongly encouraged.
